# Supplementary material for: Assessment of Iodine and Selenium Nutritional Status in Women of Reproductive Age in Latvia
Source: Medicina (Kaunas). 2021 Nov 5;57(11):1211. doi: 10.3390/medicina57111211 (PMC8622847; doi:10.3390/medicina57111211)
Supplement: Supplementary file 1 [file medicina-57-01211-s001.zip › medicina-1453008-supplementary.pdf]

**Table S1.** Main sources of iodine and selenium in women of reproductive age according to the population-based health surveys in Latvia by the survey year and age groups

| Year of the Survey Age, years | N | Proportion of respondents, % (95%CI) | | | | | | | | |
|---|---|---|---|---|---|---|---|---|---|---|
| | | Iodine | | Iodine & Selenium | Selenium | | | | | Vitamins and supplements |
| | | Use of iodized salt | At least two servings of milk or dairy products a day | Fish at least once a week | Either meat or meat products at least three days a week | Eggs at least three days a week | Rice or pasta at least three days a week | Porridges or breakfast cereal at least three days a week | At least four slices of any bread daily | Any, during the last week |
| 2010 | | | | | | | | | | |
| 15-24 | 272 | 9.6 (6.6-13.6) | 42.2 (36.4-48.2) | 47.8 (41.9-53.7) | 66.2 (60.4-71.5) | 22.4 (17.9-27.8) | 16.5 (12.6-21.4) | 19.5 (15.2-24.6) | 68.9 (63.1-74.1) | 30.5 (25.4-36.2) |
| 25-29 | 141 | 14.9 (9.9-21.7) | 41.2 (33.3-49.6) | 46.8 (38.8-55.0) | 75.9 (68.2-82.2) | 33.3 (26.1-41.5) | 16.3 (11.1-23.3) | 26.2 (19.7-34.1) | 69.1 (61.0-76.2) | 34.8 (27.4-42.9) |
| 30-34 | 163 | 20.2 (14.8-27.1) | 39.9 (32.7-47.5) | 62.0 (54.3-69.1) | 70.6 (63.1-77.0) | 42.3 (35.0-50.0) | 25.8 (19.7-33.0) | 27.6 (21.3-34.9) | 61.7 (54.0-68.9) | 35.0 (28.1-42.6) |
| 35-49 | 480 | 12.9 (10.2-16.2) | 40.1 (35.8-44.6) | 62.7 (58.3-66.9) | 69.6 (65.3-73.5) | 38.8 (34.5-43.2) | 18.1 (14.9-21.8) | 22.7 (19.2-26.7) | 70.0 (65.7-73.9) | 30.2 (26.3-34.5) |
| Total | 1056 | 13.4 (11.5-15.6) | 40.8 (37.8-43.8) | 56.6 (53.6-59.6) | 69.7 (66.9-72.4) | 34.4 (31.6-37.3) | 18.7 (16.4-21.1) | 23.1 (20.7-25.7) | 68.3 (65.4-71.1) | 31.6 (28.9-34.5) |
| 2012 | | | | | | | | | | |
| 15-24 | 275 | 6.6 (4.2-10.1) | 47.6 (41.8-53.5) | 44.7 (39.0-50.6) | 72.7 (67.2-77.7) | 34.9 (29.5-40.7) | 19.6 (15.4-24.7) | 26.9 (22.0-32.4) | 65.5 (59.7-70.8) | 27.6 (22.7-33.2) |
| 25-29 | 146 | 13.0 (8.5-19.4) | 50.7 (42.7-58.7) | 60.3 (52.2-67.9) | 75.3 (67.8-81.6) | 43.2 (35.4-51.3) | 23.3 (17.2-30.8) | 29.5 (22.7-37.3) | 72.6 (64.9-79.2) | 37.0 (29.6-45.1) |
| 30-34 | 152 | 12.5 (8.2-18.7) | 48.0 (40.2-55.9) | 64.5 (56.6-71.6) | 74.3 (66.9-80.6) | 42.1 (34.5-50.1) | 19.1 (13.6-26.1) | 28.3 (21.7-35.9) | 64.5 (56.6-71.6) | 36.2 (29.0-44.1) |
| 35-49 | 484 | 8.5 (6.3-11.3) | 47.4 (43.0-51.9) | 58.1 (53.6-62.4) | 71.5 (67.3-75.3) | 42.1 (37.8-46.6) | 21.5 (18.1-25.4) | 26.9 (23.1-31.0) | 76.2 (72.2-79.8) | 32.4 (28.4-36.7) |
| Total | 1057 | 9.2 (7.6-11.1) | 48.0 (45.0-51.0) | 55.8 (52.8-58.8) | 72.8 (70.0-75.4) | 40.4 (37.5-43.4) | 20.9 (18.6-23.5) | 27.4 (24.8-30.2) | 71.2 (68.4-73.9) | 32.4 (29.6-35.2) |
| 2014 | | | | | | | | | | |
| 15-24 | 218 | 8.3 (5.3-12.7) | 40.8 (34.5-47.5) | 46.3 (39.8-53.0) | 73.4 (67.2-78.8) | 35.3 (29.3-41.9) | 29.4 (23.7-35.7) | 22.9 (17.8-29.0) | 69.7 (63.3-75.4) | 25.2 (19.9-31.4) |
| 25-29 | 153 | 12.4 (8.1-18.6) | 37.9 (30.6-45.8) | 56.2 (48.3-63.8) | 78.4 (71.3-84.2) | 38.6 (31.2-46.5) | 25.5 (19.2-32.9) | 28.1 (21.6-35.7) | 68.0 (60.2-74.9) | 26.8 (20.4-34.3) |
| 30-34 | 162 | 11.1 (7.1-16.9) | 43.2 (35.8-50.9) | 61.1 (53.4-68.3) | 82.1 (75.5-87.2) | 46.9 (39.4-54.6) | 19.8 (14.3-26.6) | 28.4 (22.0-35.8) | 63.6 (55.9-70.6) | 32.1 (25.4-39.6) |
| 35-49 | 465 | 9.2 (6.9-12.2) | 41.3 (36.9-45.8) | 63.2 (58.7-67.5) | 76.1 (72.0-79.8) | 43.9 (39.4-48.4) | 23.4 (19.8-27.5) | 25.8 (22.0-30.0) | 71.6 (67.3-75.5) | 27.1 (23.3-31.3) |
| Total | 998 | 9.8 (8.1-11.8) | 41.0 (38.0-44.1) | 58.1 (55.0-61.1) | 76.9 (74.1-79.4) | 41.7 (38.7-44.8) | 24.4 (21.9-27.2) | 26.0 (23.3-28.8) | 69.3 (66.4-72.1) | 27.5 (24.8-30.3) |
| 2016 | | | | | | | | | | |
| 15-24 | 277 | 5.4 (3.3-8.7) | 45.5 (39.7-51.4) | 39.4 (33.8-45.2) | 69.0 (63.3-74.1) | 39.4 (33.8-45.2) | 22.4 (17.9-27.7) | 20.2 (15.9-25.3) | 60.6 (54.8-66.2) | 24.2 (19.5-29.6) |
| 25-29 | 158 | 8.2 (4.9-13.6) | 51.3 (43.5-58.9) | 51.9 (44.2-59.6) | 73.4 (66.0-79.7) | 43.7 (36.2-51.5) | 20.3 (14.7-27.2) | 34.8 (27.8-42.5) | 45.6 (38.0-53.4) | 34.8 (27.8-42.5) |
| 30-34 | 190 | 8.4 (5.2-13.2) | 44.7 (37.8-51.8) | 55.8 (48.7-62.7) | 78.4 (72.0-83.7) | 42.6 (35.8-49.7) | 20.5 (15.4-26.8) | 25.8 (20.1-32.5) | 51.1 (44.0-58.1) | 25.8 (20.1-32.5) |
| 35-49 | 542 | 9.4 (7.2-12.2) | 46.5 (42.3-50.7) | 59.4 (55.2-63.5) | 67.3 (63.3-71.2) | 45.4 (41.2-49.6) | 17.3 (14.4-20.8) | 24.4 (20.9-28.1) | 55.5 (51.3-59.7) | 32.1 (28.3-36.2) |
| Total | 1167 | 8.1 (6.7-9.9) | 46.6 (43.8-49.5) | 53.0 (50.2-55.9) | 70.4 (67.7-72.9) | 43.3 (40.5-46.1) | 19.5 (17.3-21.8) | 25.0 (22.6-27.6) | 54.7 (51.8-57.5) | 29.6 (27.0-32.2) |
| 2018 | | | | | | | | | | |
| 15-24 | 247 | 3.2 (1.6-6.3) | 30.4 (25.0-36.4) | 48.2 (42.0-54.4) | 61.9 (55.7-67.8) | 34.0 (28.4-40.1) | 35.2 (29.5-41.4) | 35.2 (29.5-41.4) | 40.5 (34.6-46.7) | 38.9 (33.0-45.1) |
| 25-29 | 138 | 6.5 (3.5-11.9) | 34.1 (26.7-42.3) | 63.8 (55.5-71.3) | 66.7 (58.4-74.0) | 34.1 (26.7-42.3) | 34.8 (27.3-43.0) | 36.2 (28.7-44.5) | 39.1 (31.4-47.5) | 54.4 (46.0-62.4) |
| 30-34 | 152 | 17.8 (12.5-24.6) | 32.2 (25.3-40.0) | 59.9 (51.9-67.3) | 75.0 (67.6-81.2) | 46.1 (38.3-54.0) | 28.3 (21.7-35.9) | 38.8 (31.4-46.8) | 40.8 (33.3-48.7) | 49.3 (41.5-57.2) |
| 35-49 | 454 | 10.8 (8.3-14.0) | 35.2 (31.0-39.7) | 60.8 (56.2-65.2) | 73.6 (69.3-77.4) | 47.8 (43.2-52.4) | 22.2 (18.7-26.3) | 30.6 (26.5-35.0) | 51.8 (47.2-56.3) | 51.1 (46.5-55.7) |
| Total | 991 | 9.4 (7.7-11.4) | 33.4 (30.5-36.4) | 57.9 (54.8-61.0) | 69.9 (67.0-72.7) | 42.2 (39.1-45.3) | 28.2 (25.4-31.0) | 33.8 (30.9-36.8) | 45.5 (42.4-48.6) | 48.2 (45.1-51.4) |
